# Dynamics of Sylvatic Chagas Disease Vectors in Coastal Ecuador Is Driven by Changes in Land Cover

**DOI:** 10.1371/journal.pntd.0002960

**Published:** 2014-06-26

**Authors:** Mario J. Grijalva, David Terán, Olivier Dangles

**Affiliations:** 1 Center for Infectious Disease Research, School of Biological Sciences, Pontifical Catholic University of Ecuador, Quito, Ecuador; 2 Tropical Disease Institute, Department of Biomedical Sciences, Heritage College of Osteopathic Medicine, Ohio University, Athens, Ohio, United States of America; 3 Laboratory of Entomology, School of Biological Sciences, Pontifical Catholic University of Ecuador, Quito, Ecuador; 4 Institut de Recherche pour le Développement (IRD), UR 072, Laboratoire Evolution, Génomes et Spéciation, UPR 9034, Centre National de la Recherche Scientifique (CNRS), Gif sur Yvette, France et Université Paris-Sud 11, Orsay, France; 5 Instituto de Ecología, Universidad Mayor San Andrés, Cotacota, La Paz, Bolivia; Universidad de Buenos Aires, Argentina

## Abstract

**Background:**

Chagas disease is a serious public health problem in Latin America where about ten million individuals show *Trypanosoma cruzi* infection. Despite significant success in controlling domiciliated triatomines, sylvatic populations frequently infest houses after insecticide treatment which hampers long term control prospects in vast geographical areas where vectorial transmission is endemic. As a key issue, the spatio-temporal dynamics of sylvatic populations is likely influenced by landscape yet evidence showing this effect is rare. The aim of this work is to examine the role of land cover changes in sylvatic triatomine ecology, based on an exhaustive field survey of pathogens, vectors, hosts, and microhabitat characteristics' dynamics.

**Methodology and Principal Findings:**

The study was performed in agricultural landscapes of coastal Ecuador as a study model. Over one year, a spatially-randomized sampling design (490 collection points) allowed quantifying triatomine densities in natural, cultivated and domestic habitats. We also assessed infection of the bugs with trypanosomes, documented their microhabitats and potential hosts, and recorded changes in landscape characteristics. In total we collected 886 individuals, mainly represented by nymphal stages of one triatomine species *Rhodnius ecuadoriensis*. As main results, we found that 1) sylvatic triatomines had very high *T. cruzi* infection rates (71%) and 2) densities of *T. cruzi*-infected sylvatic triatomines varied predictably over time due to changes in land cover and occurrence of associated rodent hosts.

**Conclusion:**

We propose a framework for identifying the factors affecting the yearly distribution of sylvatic *T. cruzi* vectors. Beyond providing key basic information for the control of human habitat colonization by sylvatic vector populations, our framework highlights the importance of both environmental and sociological factors in shaping the spatio-temporal population dynamics of triatomines. A better understanding of the dynamics of such socio-ecological systems is a crucial, yet poorly considered, issue for the long-term control of Chagas disease.

## Author Summary

Globally, more than 10 million people are infected with *Trypanosoma cruzi*. The emergence and perpetuation of Chagas disease in some endemic countries, such as Ecuador, depends largely on sylvatic populations of *T. cruzi*-infected vectors that frequently infest houses after insecticide treatment thereby hampering long-term control prospects in vast geographical areas. Our study describes, for the first time in an agricultural landscape, how the temporal dynamics of sylvatic vector, host, and pathogen populations interact spatially in a farming community of coastal Ecuador. In particular, we found that land cover changes due to both farming activities and vegetation phenology affect rodent host distribution and consequently the relative abundance of vectors involved in the transmission cycle of *T. cruzi*.

## Introduction

Chagas disease is a vector-borne disease caused by a flagellated protozoan parasite (*Trypanosoma cruzi*) that affects approximately 10 million people in Latin America and the Caribbean region [1], with an increasing number of cases reported in non-endemic countries [2]. The vectors are blood-sucking reduviid bugs of the subfamily Triatominae, of which 70 of the over 140 Triatominae species described [3], [4] have been found to be naturally infected with *T. cruzi* [5]. Originally restricted to sylvatic habitats, Chagas disease began to present a risk to human health as its vectors acquired the ability to colonize and sustain populations in human dwellings, resulting in the domiciliary transmission of *T. cruzi* [6]. In addition to domiciliated species, there is increasing evidence from several countries (e.g., Bolivia [7], Ecuador [8], [9], Brazil [4], Argentina [10]) that some sylvatic individuals frequently colonize human habitats and may play a key role in the transmission cycle of *T. cruzi*. Moreover, recent studies conducted in Ecuador indicate that house infestation, likely originating from sylvatic areas, limits the effectiveness of insecticide-based control interventions [11]. To improve our understanding of the colonization and domiciliation processes, monitoring and understanding the dynamics of sylvatic species/individuals that may colonize anthropogenic habitats has therefore become a cornerstone issue in Chagas disease research field [3].

The temporal and spatial dynamics of vector populations in sylvatic habitats is strongly influenced by the structure of the landscape and its evolution (either under natural or anthropogenic drivers) through time [12]–[14]. For example, in the Pantanal region of Brazil, changes in vegetation cover according to variations in multi-year flooding intensity strongly affect the availability of habitats occupied by rodent hosts, resulting in complex trypanosome transmission cycles [15]. Consequently, land cover modifications (e.g. ranching extension, deforestation) may affect the role that small mammals play in the transmission cycle of trypanosomes [16]. More generally, anthropogenic and natural habitat modifications increase the contact between wildlife and domestic animals, or vectors and hosts, and modify the epidemiological profiles of transmission cycles, with potential consequences on disease emergence or re-emergence patterns [17]. Moreover, the transmissibility pathways of multiple host parasites, as it is the case for *T. cruzi*, may change according to any factor affecting the population dynamics of their hosts [16]. Monitoring the sylvatic transmission cycles of these trypanosomatids is therefore necessary to prevent re-emergence of Chagas disease.

The objective of this work was to assess whether temporal changes in land cover characteristics may affect the spatial distribution and host association of *T. cruzi* vectors. The study was performed in agricultural landscapes of coastal Ecuador as a study model. In this country, around 3.8 million people are at risk of acquiring Chagas disease and some 200,000 individuals are currently infected with the disease, making it a serious public health issue [18]. Over one year, we sampled triatomine individuals in natural (forest or shrub), cultivated and domestic habitats; assessed their infection with trypanosomes; and recorded changes in landscape characteristics. As the distribution of sylvatic triatomine species has been found to be related to the presence of rodent hosts (mainly mice, rats and squirrels [8]) themselves influenced by vegetation type and cover, we hypothesized that temporal land cover changes partly related to the crop production cycle (planting, harvesting, absence of crop) may affect the presence of rodent hosts and consequently that of associated triatomines.

## Materials and Methods

### Ethics Statement

We obtained informed consent from the head of the household following protocols approved by the institutional review boards of Ohio University and Catholic University of Ecuador (PUCE).

### Study Site

Our study was conducted in the rural community of *El Bejuco* located in Portoviejo County, Manabí Province (0°57′37.80″S; 80°14′0.51″W) in northwestern Ecuador. This community was chosen for the high triatomine baseline and post-insecticide spraying household infestation [11] and the presence of a landscape composed of both natural and cultivated areas. This community comprised 98 houses with an approximate number of 450 inhabitants. Most houses are constructed with bamboo walls, concrete bricks and timber with roofs made of corrugated metal sheets or *Phytelephas aequatorialis* Spruce palm leaves [19]. The peridomestic areas contained dogs, chickens, pigs and cows dwelling among firewood piles and trees [11]. Average monthly temperatures range from 18.7 to 30.8°C with a mean value of 24.8°C. The precipitation regime is characterized by two main seasons: 1) a rainy season (total precipitation = 779 mm) between January and April and 2) a dry season (total precipitations = 26 mm) between July and October (data from the WorldClim data set). The average relative humidity ranges between 90 and 97% over the year [8].

### Triatomine Sampling

**Figure 1:**
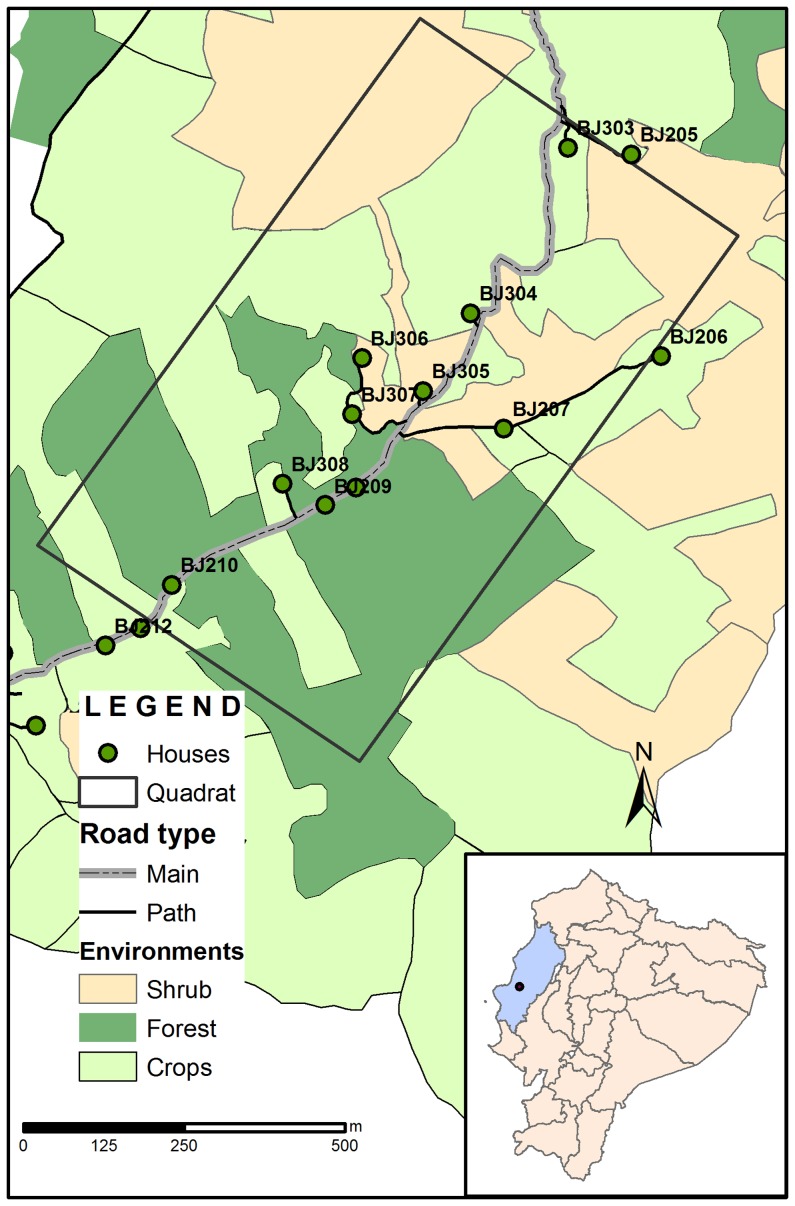
Map of the study area indicating the location of the quadrat, digital representation of cultivated areas [crops], and natural vegetation [forest or shrub]) and an overlay of the road and house locations. Insert indicates the location of the study area within the political map of Ecuador.

Our sampling area in the community was defined as a 1000 m×600 m quadrat located along the main road of the village (Figure 1). This quadrat included the different environments found in the community (synanthropic [defined as peridomestic and domestic habitats], cultivated areas [crops], and natural vegetation [forest or shrub]). Sampling was performed on seven visits from June 2009 to June 2010, with intervals of two months between visits. Two visits (February and April) were performed during the rainy season, two (August and October) during the dry season, and three (June and December 2009, and June 2010) during the transition periods. Triatomine sampling in synanthropic environments (12 houses located in the sampling area) was conducted on every visit by a three-person team using standardized protocols during timed manual collections as previously described [20]. If at least one live triatomine was found in a domestic unit (DU), this house was classified as being infested and was sprayed by trained field workers from the Ministry of Health with deltamethrin at a rate of 25 mg a.i per m^2^ as previously described [11]. Spraying included all structures found in the domicile and peridomicile and were supervised by staff from PUCE. Houses found to be not-infested were not sprayed.

Sylvatic populations of triatomines (defined as populations sampled in both cultivated and natural vegetation) were sampled following the randomized sampling design developed by Suarez-Davalos et al. [8]. During each visit the coordinates of 70 points were randomly generated within the quadrat using the Spatstat package of R Software (R Development Core Team 2012). In the case that the distance between two points was <3 m, the whole randomization was rerun (see [8] for more details). For all dates, the minimal distance between each collection point was 10.2 m. These coordinates were then transferred to a GPS receiver (Meridian Platinum, Magellan, San Dimas, United States) for field sampling. At each point, standard manual searches for triatomines were performed for 30 min by three-person teams in different microhabitats (nests, burrows, tree holes, and under trunks and rocks) within a three-meter radius area [21]. When palms were found within the sampling radius, manual searches were conducted by skilled personnel. Briefly, one skilled searcher used a ladder to perform the search from the base of the leaves. Manual searches for triatomines were carried out within the foliage. Any nest found was carefully extracted and dissected on a 2 m^2^ yellow plastic sheet placed at the base of the palm. Underground mammal burrows were searched by extracting all material found within placed onto a yellow plastic sheet. The total area sampled on each occasion was 1981 m^2^. In each microhabitat, we recorded information regarding habitat type (forest, shrub, or crop; see below), occurrence of vertebrate hosts (identified to species level based on nest occurrence), and nest height from ground level. At each date, sampling lasted for 5 days, representing a total sampling effort of 840 person-hours for the whole study.

### Landscape Features and Dynamics

About 50% of the total land cover of the studied community was composed of cultivated areas, mainly corn, papaya, and peanut crops. The rest of the landscape was vegetated with a lowland semideciduous forest composed of thorny plants and trees that lose their leaves once a year (e.g., *Ceratonia siliqua* L. 1858 and *Guazuma ulmifolia* Lam. 1789) [8]. In this region, there is only one harvest of short-cycle maize per year as there is not enough precipitation during the dry season and most farmers do not have irrigation facilities. Maize is generally planted during the rainy season and is harvested during the following transition period (May–June).

For the seven sampling periods, each of the 70 sampled points was assigned to a habitat type as follows: 1) forest, characterized by a high density of *G. ulmifolia* tree, 2) shrub, characterized by high density of *Cordia lutea* Lam. and 3) crops. While elaborated landscape analyses can be performed using indices describing the landscape based on the composition and disposition of its constituents [22], we realized that, at our study scale, a simple characterization of land cover modification over the whole year at each of the 490 triatomine sampling points (70 sampling points×7 months) and 12 houses was sufficient to describe the dynamics of this agricultural landscape [23].

### Triatomine Identification and *T. cruzi* Analyses

Once collected, triatomines were placed in individually labeled plastic containers and transported to the Center for Infectious Disease Research (PUCE, Quito) where they were counted and identified to the species and instar level. Species identification was based on morphological criteria [24], [25] and comparisons with pinned specimens of the PUCE's Entomology Museum (QCAZ). The presence of *T. cruzi*-like organisms was determined by examination of the feces and intestinal content at ×400 magnification, using a direct light microscope and by PCR using S35/S36 and 121/122 primer sets [26], [27]. Discrete typing units (DTU) were determined as described in Ocaña-Mayorga et al. [28].

### Data Analyses

#### Triatomine infestation

Four entomological indices were calculated to describe triatomine infestation levels in both synanthropic and sylvatic environments over the 7 sampling dates: (1) infestation index (number of positive houses or sylvatic collection point/number of houses or sylvatic collection points examined×100), (2) density (number of triatomines collected/number of houses or nests examined), (3) crowding (number of triatomines collected/number of positive houses or nests), and (4) colonization index (number of houses or nests with nymphs/number of positive houses or nests×100) [11], [29].

#### Triatomine density interpolation mapping

To visualize the spatio-temporal dynamics of triatomine abundance in the studied community we built density interpolation maps using information on the number of triatomines collected at the coordinates of the 70-quadrat points at each visit. The data were adjusted at a surface z(x,y) form, by means of the Gridfit function of the Software MATLAB 7.0 [30]. Interpolations relating information between nearest neighbors were obtained to develop a Gridfit-modeled surface compiled with the plot of the sampled points (see Dangles et al. [31] for more details). These plots were overlapped with maps of synanthropic infestation generated in ArcMap 9.3 (ESRI Inc. 1999–2008).

#### Multivariate and path analyses

We used ordination techniques coupled with path analyses to examine the direct and indirect effects of season (reflected by precipitation levels) on *T. cruzi*-infected abundance. First, in order to test whether landscape changes over the year (i.e. 7 sampling dates) affected the distribution of triatomines, multivariate analysis of variance (MANOVA) and canonical variate analyses (CVA) were performed on habitat type variables (crop, shrub, forest) and rodent host variables (squirrel, rat and mouse) associated with the locations where triatomines were found. This analysis is analogous to a principal component of the main directions of variation among dates and allowed us to quantify the environmental spaces of infected triatomines and their temporal variation. Non-parametric MANOVA (10,000 permutations, p-values Bonferroni corrected) was performed on the Euclidean distances among pair of points [32]. The Wilks' k from the MANOVA represents the ratio between pooled within-groups and total covariance matrices. Values close to 1.0 indicate equality of covariance matrices within and among dates and, thus, no statistical differences among their centroids. CVA results were cross-validated using the leave-one-out procedure (Jackknife procedure) on the classifier matrix, where a concordance between classification success rates in the non-validated and validated analyses indicates that group discrimination was not based on a one-case contribution. All these analyses were run in PAST 2.0 using both total triatomine and *T. cruzi*-infected triatomine. As both analyses gave similar results, only those concerning infected triatomines are presented.

Second, we used path analysis via structural equation modeling as a powerful method exploring causal mechanisms that link triatomine abundance to environmental changes over one year [33]. Based on prior ecological knowledge on Ecuadorian triatomines in agricultural landscapes [8], [20], [21], [34], we used a relatively simple model in which the dependent variable was the abundance of *T. cruzi*-infected triatomine while the independent variable was season, expressed as rainfall. We used this model to assess how strong the season effect was on triatomine abundance and whether it was acting directly or indirectly through other factors such as habitat availability (either crop or shrubs and forest) and host occurrence (mice, rats and squirrels). More complex models were not used because sample size would not support them. All variables were transformed to have a normal distribution using *z* scores and log transformation prior to analysis. Path analyses were run in the sem package [35] for R, version 2.15.1 (R Development Core Team 2013).

#### Generalized linear model

Generalized linear model analyses (GLM) were further used to test the effect of specific environmental variables on the density of triatomines. The variables included in the Poisson log-linear models were: date, habitats (forest, shrub, and crop), host (mouse, rat and squirrel), nest height, and the number of houses sprayed at the preceding sampling date. To avoid multicollinearity, some of the environmental variables that were highly correlated (| r |>0.7) (e.g. nest height and host) were not included in the same model. We also included a “collection point” variable to allow within-collection point comparisons while controlling for variation resulting from unmeasured collection point-specific parameters. Site was treated as fixed effects, but we obtained similar results by means of the generalized linear mixed model function (glmmPQL; MASS library for R), with sites as random effects. Change in triatomine densities due to each factor was modeled considering each factor independently and in combination with other factors, including biologically reasonable two-way interactions, and squared variables. The more parsimonious model was identified using the Akaike's Information Criterion (AIC, see [36]) in likelihood ratio tests to find the difference between the initial model and the reduced model, dropping an “effect” term. All analyses were performed on log-transformed data in R [37] using both total triatomine and *T. cruzi*-infected triatomine. As both analyses gave similar results, only those concerning infected triatomines are presented.

## Results

A total of 886 triatomine specimens were collected over the seven sampling dates. Ninety nine percent of all collected specimens were *Rhodnius ecuadoriensis* Lent & León, 1958. The remaining five specimens were *Panstrongylus howardi* Neiva 1911 (3 individuals) collected in a squirrel nest in June 2009, one, *P. geniculatus* Latreille 1811 (1 individual) collected in the peridomicile in June 2009 and one *P. rufotuberculatus* collected in a squirrel nest in December 2009. Of the 886 specimens, 69% were collected in sylvatic habitats. As a general pattern, we collected a high number of first instar nymphs but few adults of *R. ecuadoriensis* in both sylvatic and synanthropic habitats. The majority (78.1%) of sylvatic triatomines were found in animal nests. These nests were either of squirrel (*Sciurus stramineus* Eydoux & Souleyet 1841, 83.1%), mouse/rat (10.9% e.g *Sigmodon sp, Proechimys sp, Rhipidomys sp, Mus musculus* L.,), bird (4.9%, e.g., *Campylorhynchus fasciatus* Swainson 1837) or other species (e.g., the opossum *Didelphis marsupialis* L. 1858, 1.1%). While triatomines were commonly found in squirrel nests all year round, they were associated with mouse/rat nests mostly during the rainy and transition seasons. Triatomines were not found in an abandoned structure, which was the only bat habitat found within the quadrat.

**Figure 2:**
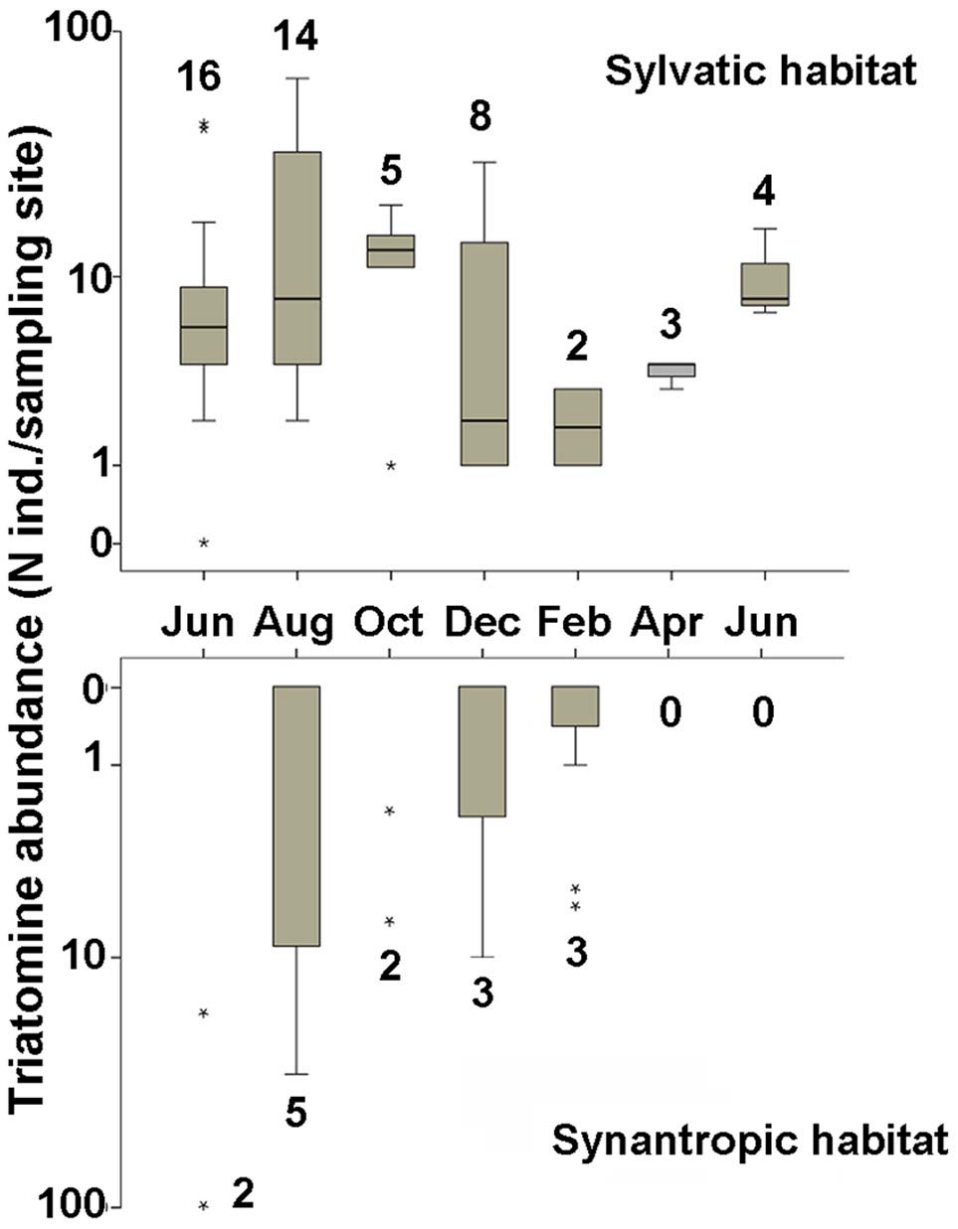
Box-plot representation of abundance of triatomines expressed as the number of individuals collected by collection point in both sylvatic and domestic habitats for the 7 sampling dates. Number above/below the box-plots, indicate the number of infested points. Box-plots were built using data from the 70 sampled sylvatic points in the quadrat and the seven infested houses (see Table S1).

The infestation index ranged from 4.3% to 22.9% and 0% to 41.7% in sylvatic and synanthropic habitats, respectively (Table 1). The density index was generally lower in sylvatic than synanthropic habitats (except in April and June 2010) while the crowding index greatly varied among dates in both habitats. Colonization indices were always >85% in both habitats (except in December, April and June in houses). As a general pattern, the value of the three first entomological indices tended to be low for the last sampling dates (February to June 2010). This tendency was consistent with lower triatomine abundances and number of infested points found at the end of the sampling period (see Figure 2). Overall, half of the 12 sampled houses presented triatomine infestation at least once during the year-long survey. Of those, five houses had multiple infestations at different time points (Table S1).

**Table 1:** Entomological indices of Triatomine (*R. ecuadoriensis*) infestation calculated for both synanthropic (domestic-peridomestic, 12 DUs at each visit) and sylvatic habitats (70 sampling point at each visit) over the seven field visits.

		Jun	Aug	Oct	Dec	Feb	Apr	Jun
Infestation index (%)	Sylvatic	22.9	20.0	7.1	11.4	2.9	4.3	5.7
	Synanthropic	16.7	41.7	16.7	33.3	25.0	0.0	0.0
Density index	Sylvatic	2.5	3.7	0.9	1.0	0.1	0.2	0.4
	Synanthropic	8.0	5.4	0.8	7.7	1.4	0.0	0.0
Crowding index	Sylvatic	10.9	18.6	12.0	8.9	2.0	3.7	7.8
	Synanthropic	48.0	13.0	4.5	23.0	5.7	0.0	0.0
Colonization index (%)	Sylvatic	93.6	100.0	100.0	87.5	100.0	100.0	100.0
	Synanthropic	100.0	100.0	100.0	50.0	100.0	0.0	0.0

Triatomine abundance for each sampling date can be seen in Figure 2.

**Figure 3:**
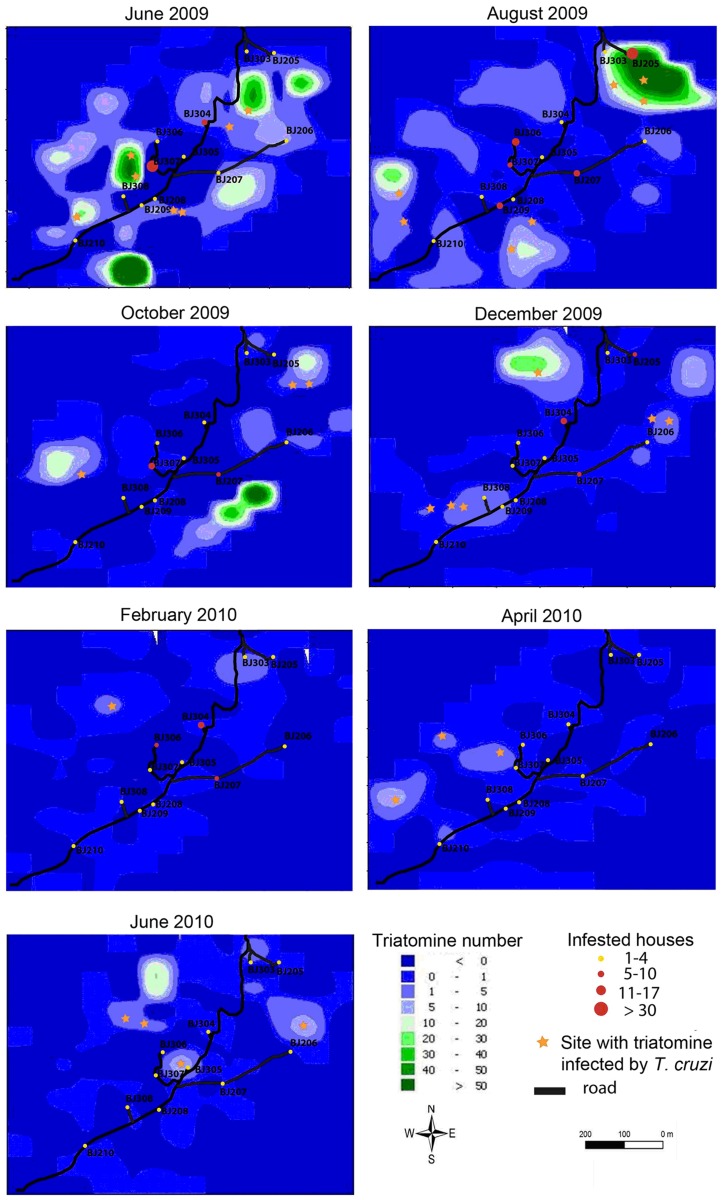
Triatomine density interpolation maps of the study community for the 7 sampling dates. Maps are based on data from both sylvatic and domestic habitats. Black lines represent principal and secondary roads in the community. Circles indicate houses that were visited. Orange stars represent collection points where triatomines infected by *T. cruzi* were sampled.

Overall, triatomine abundance was highly variable among collection points at a given date (see Figure 2), resulting in the observed aggregated pattern of triatomine spatial distribution (Figure 3). Interestingly, these “hotspots” of triatomine density were located both nearby (<50 m) and relatively far (>200 m) from the houses.

**Figure 4:**
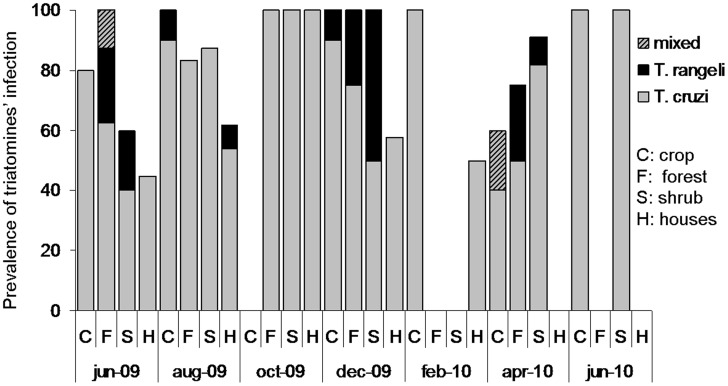
Prevalence of triatomines' infection by *T. cruzi*, *T. rangeli* and mixed infections for each sampling date and habitat. X axis labels correspond to C = crops, F = forest, S = shrub and H = House.

Infections with *Trypanosoma* spp were detected by PCR in 90% of the 105 sylvatic triatomines analyzed. Infections with *T. cruzi* were the most common (71%) followed by infections with *T. rangeli* (15%) and mixed infections *T. cruzi*/*T. rangeli* (4 cases, 4%). Infection rates were lower for individuals sampled in houses (60%, N = 55) with a large predominance of *T. cruzi* infections (58%), the remaining being infected by *T. rangeli*. The prevalence of triatomines' infection for each date and habitat is given in Figure 4.

**Figure 5:**
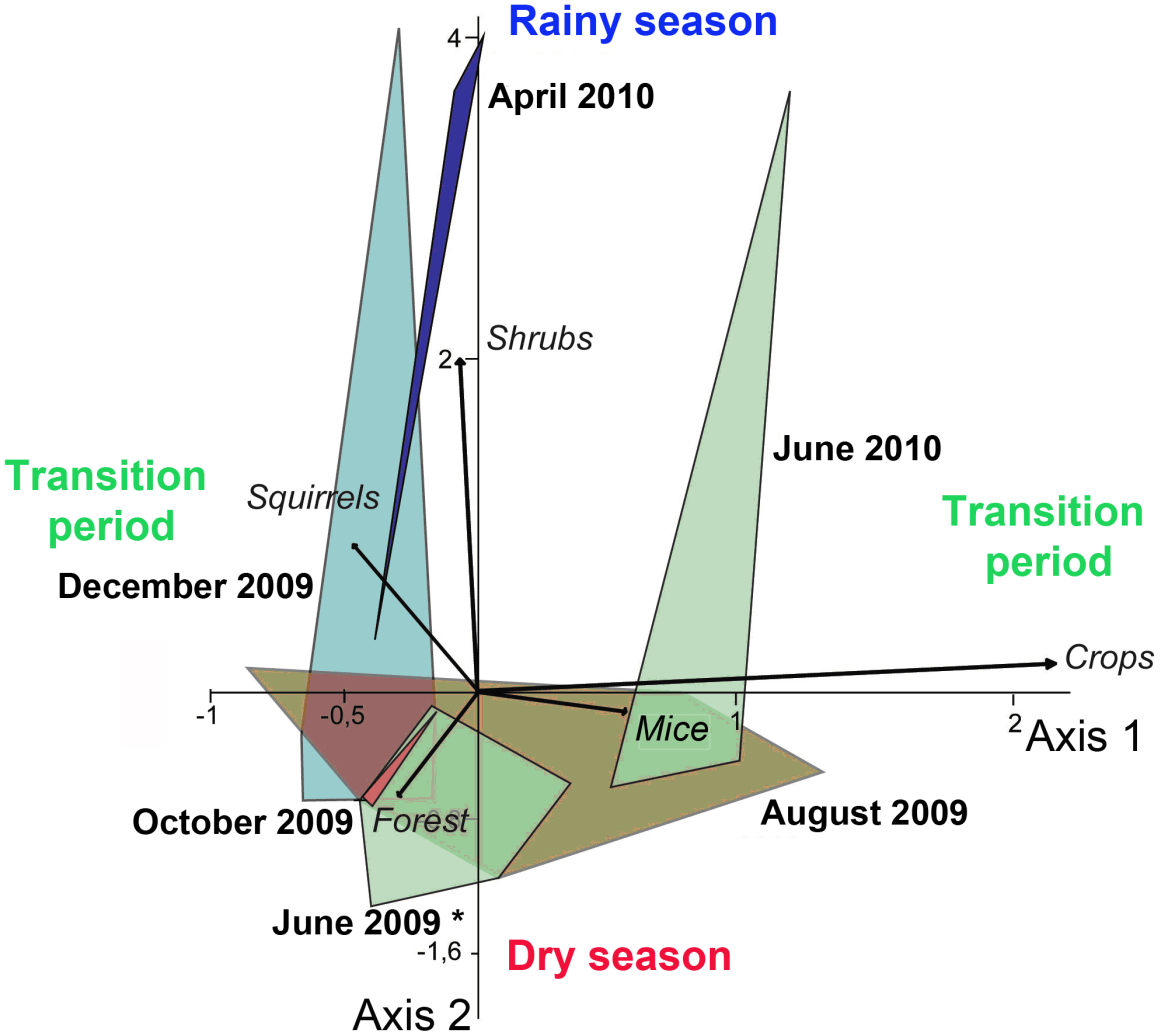
Canonical ordination of the environmental variables associated with the presence of *T. cruzi*-infected triatomines for the different sampling dates between June 2009 and June 2010. Polygons represent the “environmental spaces” (see main text) of infected triatomines at each date. The first canonical axis explains 64.7% of the variation among groups.

**Figure 6:**
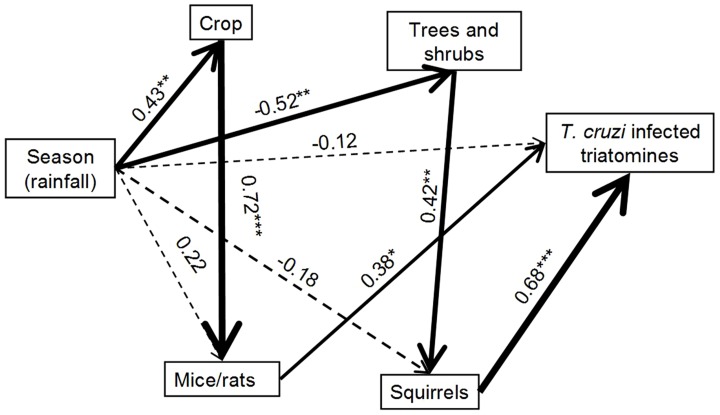
Path diagram resulting from structural equation modelling of factors affecting populations of *T. cruzi*-infected triatomines in Manabi, Coastal Ecuador (Bentler CFI = 0.91). Path coefficient and significance (* P≤0.05; ** P≤0.01; *** P≤0.001) are given above each arrow. Arrow thickness is scaled by the standardized loading coefficients for each connection. Dashed arrows are non significant.

Multivariate analyses (MANOVA and CVA) showed that the environmental spaces of *T. cruzi*-infected triatomines were significantly different among dates (Wilks' k from MANOVA = 0.119; P<0.001). The first canonical variate (axis 1), primarily associated with crop and mice, explained 64.7% of the variability among dates and clearly discriminated the environmental spaces from dry (August–October) to rainy season (April; Figure 5). The second canonical variate (axis 2, 23.7%) was primarily associated with shrubs and tended to separate the transition season following the dry season (December) from the transition season following the rainy season (June; Figure 5). Note that May–June 2009 experienced unusually low precipitation, which explains the position of its environmental space together with August and October. Path analyses further suggested that rainfall significantly determined the abundance of *T. cruzi*-infected triatomines via indirect paths involving habitats and rodent hosts (Figure 6). Rainfall had a significantly positive and negative influence on crop and forest/shrub cover, respectively. In turn, these habitats significantly influence the occurrence of rodent hosts and thereby the abundance of *T. cruzi*-infected triatomines.

Of the five factors included in the GLM analysis (see Materials and Methods) three of them, date, habitat, and host, significantly affected the abundance of infected triatomines with ‘habitat’ and ‘date’ showing the highest ΔAIC values (Table 2). Moreover, the significant ‘date×habitat’ interaction term revealed that changes in habitat structure over the year influenced significantly the abundance of infected triatomines. We also found a significant ‘date×vector’ interaction term which supports that changes in rodent host populations affect the distribution of *T. cruzi*-infected triatomine through time. Note that similar results were found when performing the analyses with all triatomine data (infected and non-infected, Table S2).

**Table 2:** Results of the generalised linear model deviance analysis on the number N of triatomines (*R. ecuadoriensis*) infected by *T. cruzi*.

Effect	AIC	Δ AIC	LRT	*P*-value
Date	234.6	33.5	5.89	0.005
Habitat	280.1	79.0	8.42	0.001
Host	213.9	12.8	4.84	0.007
House spraying	202.3	1.5	1.84	0.079
Date×host	207.9	6.8	3.46	0.005
Date×habitat	207.0	5.9	3.34	0.006

The model was: *N∼Date+habitat+host+nest height+house spraying+date×habitat+date×host+habitat×host+nest height×habitat*. For each effect variable, Δ AIC corresponds to the difference between the AIC of the initial model and that of reduced model. Likelihood-ratio test (LRT) and associated *P*-value test the hypothesis that the suppression of the ‘effect’ term provides no better fit than the initial model.

AIC = Akaike's information criterion of the initial model after the removal of the ‘effect’ term.

## Discussion

Our study confirms the prominent role of sylvatic triatomines in Chagas disease epidemiology due to their very high infection rates by *T. cruzi* (71%). We also provide strong evidence that environmental spaces of triatomines (either infected or not) varied predictably over the year due to changes in land cover (in particular the cultivated-natural habitat switch) and associated occurrence of rodent hosts. This highlights the importance of both environmental and sociological factors (e.g., agricultural scheduling) in shaping the spatio-temporal population dynamics of triatomines.

### Triatomine Spatio-temporal Dynamics

Our study reports that the species *R. ecuadoriensis*, the most important Chagas disease vector in coastal Ecuador, has well established *T. cruzi*-infected sylvatic populations all year round. Although the occurrence of these vectors in Ecuador is well known [8], [34], [38], no detailed data on their temporal dynamics were available. Our findings confirm that the demographic structure of sylvatic *R. ecuadoriensis* population was dominated by nymphal stages over the year [8] and that aggregations occurred both in sylvatic habitats and closer to homes [8], [39]. In agricultural landscapes such as coastal Ecuador, the impact of human activities through crop planting and deforestation can extend far beyond the close periphery of households, which may explain the presence of triatomine aggregation areas relatively far from populated areas [40], [41]. As previously reported by [14], these results emphasize the importance of sylvatic populations for the transmission cycle of *T. cruzi*. Currently there are no genetic markers available to conduct population genetic studies that compare populations of *R. ecuadoriensis* collected in houses and sylvatic environments. However, morphometric analyses using antennae sencilla and wing geometry failed to detect significant differences between these populations [42].

As found in other studies (e.g., Mexico [23], USA [43]), triatomines were strongly associated with squirrels which probably represent one of the most important host species [21], [34]. The relatively large body size of squirrels and the characteristics of their nests (large, 30 cm diameter, loose construction, and usually located at >5 m from the ground) could improve food source availability and protection for triatomines [8]. The spatio-temporal dynamics of triatomine bugs in our study area was therefore highly influenced by variations in the occurrence of squirrels over the year and their distribution among the different habitat types (see below). These results confirm a previous study by Gottdenker et al. [12], [13] who found that changing host community structure following anthropogenic landscape disturbance in Panama may increase vector infection with *T. cruzi*. These authors further showed the importance of host reproductive rates as an important determinant of vector infection, suggesting that further research on rodent host life history in coastal Ecuador would be needed to improve our knowledge on *T. cruzi* epidemiology.

### *T. cruzi*-Infected Sylvatic Triatomine Cycle

Several studies have been performed to characterize and correlate sylvatic and synanthropic habitats with triatomine presence/absence using environmental parameters such as climate and topography [44], [45], associations with mammal hosts [16], [46], birds and palm trees [9], [47]; (Grijalva unpublished data), or specific land-cover types [48]. As revealed by previous studies from other parts of Latin America [12]–[16], the spatio-temporal distribution cycle of *R. ecuadoriensis* in coastal Ecuador comes as a result of a combination of multiple ecological factors. Because climatic conditions of the Ecuadorian Coast are favorable for triatomine development all year round (all months are moist and warm), land cover features represent the key variables that shape the population dynamics of *T. cruzi* vectors on a local scale [8]. Moreover, in view of the importance of agricultural activities in coastal Ecuador, land cover patterns and dynamics are likely influenced by land use practices of rural communities [11].

**Figure 7:**
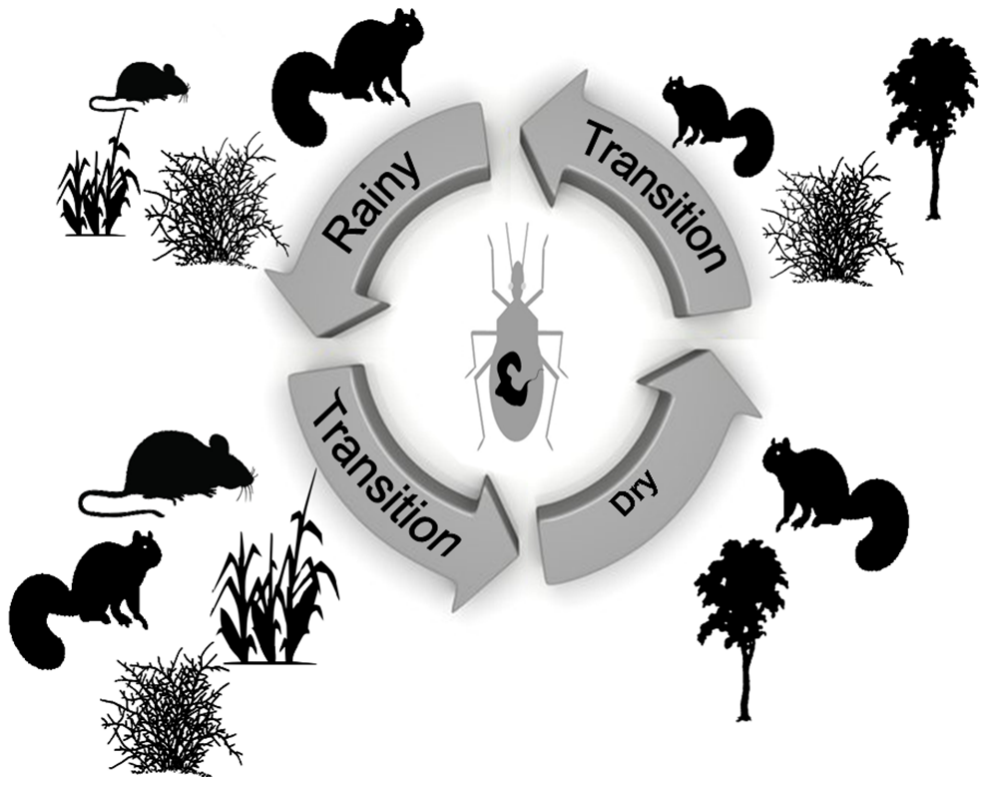
Cycle of infected sylvatic triatomines in coastal Ecuador resulting from changes in land cover (crop, forest, and shrub) and associated host distribution (mice and squirrels). The relative size of land cover and animal icons refers to the relative importance of these factors. Rainy period: Jan–Apr, Transition period 1: May–June, Dry period: July–Oct., Transition period 2 = Nov.–Dec.

Our quantitative results from both the MANOVA-CVA, the path analysis and the GLM analyses (themselves based on an extensive sampling of 886 triatomine individuals and associated micro-environmental parameters over one year) strongly suggest that variations in land cover over one year, and subsequent effect on the distribution of rodent hosts, drive *R. ecuadoriensis* population dynamics in coastal Ecuador. While, for logistical reasons, our sampling was limited to one year in one location, results found are in agreement with other studies performed by our team over the last 10 years in other regions of Ecuador at different periods of the year (e.g. [8], [11], [20], [34], [38]), reinforcing their general relevance. Based on this accumulated knowledge, we propose a schematic framework identifying the factors affecting the distribution of sylvatic *T. cruzi* vectors over one year (Figure 7). During the rainy season (January–April), triatomines are mainly associated with squirrels whose nests are abundant in leafy bushes, hidden from predators. Some triatomines are also found in mouse nests in growing crops. A few months later, crops mature and food availability increases the association of triatomine with mice/rats, which become important hosts for them. During the dry season (July–October) triatomines mainly colonize squirrel nests located in trees. As many tree and shrub species lose their leaves at this season, it may be more secure for squirrels to nest high in the trees, generally >5 m, [8] than in shrubs. Finally, during the transition period that follows the dry season, triatomines are only found at low densities in squirrel nests associated with tree and leafing bushes. This season seems to be the most critical for the survival of sylvatic triatomines due to poor resource conditions in term of both habitat and hosts. Overall, land cover changes due to both farming activities and vegetation phenology affect rodent host distribution and, consequently, that of triatomines. Such findings extend to agricultural systems the results of previous studies in forested habitats that have shown that the composition and relative abundance of small mammal fauna involved in the transmission cycle of Chagas disease can be influenced by landscape structure and its changes over the year [16].

Still, causal mechanisms that link triatomine abundance to seasonal environmental changes are poorly understood due to the difficulty in performing large-scale landscape manipulation experiments and evaluating contributions from direct and indirect factors that can have opposing effects on triatomine populations [13]. However, our path analysis helped to stress the importance of indirect effects from field data. The triatomine population studied here clearly responded primarily to local habitat and host availability changes, which were changing over the year due to differences in precipitation. Additional work is needed to fully understand the epidemiological cycle of *T. cruzi* in changing landscapes. Our path analysis suggests individual hypotheses that can be tested experimentally or further investigated with more detailed observations as each arrow or path identifies a putative causal relationship between the triatomine populations and seasonality-driven environmental aspects.

### Implications for Control

The burden of endemic neglected zoonoses generally falls heavily on rural communities with limited resources. In Ecuador, rural communities with subsistence-farming practices are high-risk areas for acquiring Chagas disease [20]. As control of Chagas disease should rely on the interruption of parasite transmission to domestic hosts [8], a sound understanding of infection risk factors in both sylvatic and synanthropic vector populations is needed to effectively assist the development of effective prevention programs [49]. In this context, the results of this study have three major implications. First, our study emphasizes the importance of sylvatic triatomine populations as a main component of the transmission cycle of *T. cruzi*. While insecticide spraying in houses have allowed the control of domestic individuals (see Figure 2), numerous sylvatic individuals remain in the vicinity of households, which constitute a potential source of vectors for re-colonization [11]. Second, while there is a decline in the number of individuals over time, our results corroborate the previously reported low effectiveness of deltamethrin and suggest a short lasting residual effect of deltamethrin in the environmental conditions of coastal Ecuador. This has been observed in other areas such as the Chaco region [50]. Third, our findings highlight the importance of both environmental and sociological (farming practices) factors in shaping the spatio-temporal population dynamics of *T. cruzi* vectors. We propose that further research on *T. cruzi* transmission cycles should follow a social-ecological approach (e.g. using methods developed for the study of socio-ecological systems, such as agent-based models, [51]), in which the coupling of human and natural systems would reveal the complex patterns and processes emerging from their interactions [52]. For example, if farming communities of coastal Ecuador would develop irrigation to support cultivation of maize during the dry season, this may have an impact on the sylvatic triatomine cycle through an increase of mouse/rat and squirrel occurrence during the dry and following transition seasons. However, two maize harvests per year would boost farmers' income and probably improve their living conditions, thereby decreasing their vulnerability to Chagas disease [1] and potentially reducing *T. cruzi* transmission [53]. Indeed, even low cost home improvements that limit areas of vector refuge in nearby houses can be highly effective at keeping infestation low [54]. Understanding such complex effects of land use changes on *T. cruzi* transmission and overall potential negative and/or positive feedback between farming practices, habitat availability, hosts and vectors would provide crucial, yet poorly considered, information with strong implications for vector surveillance and control.

## Supporting Information

Table S1 Number of triatomines per house collected in the 12 sampled individual houses within the quadrat over the study period (June 2009–June 2010).
(DOCX)

Table S2 Results of the generalised linear model deviance analysis on the total number *N* of triatomines (*R. ecuadoriensis*). AIC = Akaike's information criterion of the initial model after the removal of the ‘effect’ term.
(DOCX)
