# Supplementary material for: Dynamics of Sylvatic Chagas Disease Vectors in Coastal Ecuador Is Driven by Changes in Land Cover
Source: PLoS Negl Trop Dis. 2014 Jun 26;8(6):e2960. doi: 10.1371/journal.pntd.0002960 (PMC4072561; doi:10.1371/journal.pntd.0002960)
Supplement: Table S1 — Number of triatomines per house collected in the 12 sampled individual houses within the quadrat over the study period (June 2009–June 2010). (DOCX) [file pntd.0002960.s001.docx]

**Table S1.** Number of triatomines per house collected in the 12 sampled individual houses within the quadrat over the study period (June 2009 - June 2010).

| House Code | Jun | Aug | Oct | Dec | Feb | Apr | Jun |
| --- | --- | --- | --- | --- | --- | --- | --- |
| BJ205 | - | 30 | - | 4 | - | - | - |
| BJ206 | - | - | - | - | - | - | - |
| BJ207 | - | 10 | 2 | 4 | 1 | - | - |
| BJ208 | - | - | - | - | - | - | - |
| BJ209 | - | 8 | - | - | - | - | - |
| BJ210 | - | - | - | - | - | - | - |
| BJ303 | - | - | - | - | - | - | - |
| BJ304 | 17 | - | - | 10 | 5 | - | - |
| BJ305 | - | - | - | 1 | - | - | - |
| BJ306 | - | 13 | - | 1 | 6 | - | - |
| BJ307 | 109 | 5 | 7 | - | - | - | - |
| BJ308 | - | - | - | - | - | - | - |

Dashes denote the absence of infestation. The House codes refer to figure 3.
