# Supplementary material for: Dynamics of Sylvatic Chagas Disease Vectors in Coastal Ecuador Is Driven by Changes in Land Cover
Source: PLoS Negl Trop Dis. 2014 Jun 26;8(6):e2960. doi: 10.1371/journal.pntd.0002960 (PMC4072561; doi:10.1371/journal.pntd.0002960)
Supplement: Table S2 — Results of the generalised linear model deviance analysis on the total number N of triatomines (R. ecuadoriensis). AIC = Akaike's information criterion of the initial model after the removal of the ‘effect’ term. (DOCX) [file pntd.0002960.s002.docx]

**Table S2.** Results of the generalised linear model deviance analysis on the total number *N* of triatomines (*R. ecuadoriensis*). AIC = Akaike’s information criterion of the initial model after the removal of the ‘effect’ term.

| Effect | AIC | Δ AIC | LRT | *P*-value |
| --- | --- | --- | --- | --- |
| Date | 57.6 | 22.1 | 6.35 | 0.008 |
| Habitat | 61.5 | 26.0 | 7.32 | 0.002 |
| Host | 42.4 | 6.9 | 3.84 | 0.007 |
| House spraying | 37.2 | 1.7 | 1.93 | 0.068 |
| Date × host | 40.0 | 4.5 | 2.96 | 0.011 |
| Date × habitat | 40.4 | 4.9 | 3.01 | 0.009 |

The model was: *N ~ Date + habitat + host + nest height + house spraying + date×habitat + date×host + habitat×host + nest height×habitat*. For each effect variable, Δ AIC corresponds to the difference between the AIC of the initial model and that of reduced model. Likelihood-ratio test (LRT) and associated *P*-value test the hypothesis that the suppression of the ‘effect’ term provides no better fit than the initial model.
